# Relations of Distinct Psychopathic Personality Traits with Anxiety and Fear: Findings from Offenders and Non-Offenders

**DOI:** 10.1371/journal.pone.0143120

**Published:** 2015-11-16

**Authors:** Steven M. Gillespie, Ian J. Mitchell, Rose-Marie Satherley, Anthony R. Beech, Pia Rotshtein

**Affiliations:** School of Psychology, University of Birmingham, Birmingham, United Kingdom; University of Wisconsin-Milwaukee, UNITED STATES

## Abstract

Early descriptions of psychopathy emphasise fearlessness and a lack of nervousness or anxiety as key characteristics of the disorder. However, conflicting evidence suggests that anxiety may be positively correlated with some aspects of the psychopathy construct. This position may seem somewhat paradoxical when considered alongside impaired processing of fear related stimuli in psychopathic personality. The aim of the current paper was to examine the distinct relations of callous, egocentric, and antisocial psychopathic traits with measures of anxiety and social anxiety in samples of non-offenders (Study 1) and violent offenders (Study 2). In Study 2 we also used an emotion recognition task to examine fearful face recognition. In Studies 1 and 2 we showed distinct and opposite significant relationships of egocentric and antisocial psychopathic traits with trait anxiety. Thus, while trait anxiety was negatively predicted by egocentric traits, it was predicted in a positive direction by antisocial traits in both samples. In Study 2 we found that callous traits were predictive of greater impairments in fearful face recognition. These findings suggest that anxiety and fear are distinguishable constructs in relation to psychopathic personality traits, and are discussed in terms of potentially separable mechanisms for these two constructs.

## Introduction

Cleckley [1], in his seminal description of psychopathy, describes a distinct subgroup of psychiatric patients who beneath a façade of normalness, presented as severely callous, devoid of human emotion, and lacking in nervousness or anxiety. These criteria are largely relevant to modern day conceptualizations of psychopathy and have been adapted for use in the Psychopathy Checklist–Revised [PCL-R] [2, 3] for the measurement of psychopathic personality. The PCL-R contains two broad Factors pertaining to the clinical features of psychopathy: Factor 1 and Factor 2. While Factor 1 measures the interpersonal/affective features of psychopathy, including a callous lack of empathy and a deceitful and manipulative interpersonal style, Factor 2 taps lifestyle/antisocial features, including need for stimulation/proneness to boredom, and poor behavioural controls [3].

Relative to non-psychopaths, psychopaths are characterized by heightened levels of instrumentally violent or goal directed acts of aggression [4, 5]. This is in contrast to reactive aggression that is often in response to a perceived slight or threat and serves no instrumental purpose [6]. It is argued that psychopaths’ increased use of instrumental aggression may reflect a failure to recognize and experience others distress cues as aversive [7, 8]. In support of this model, adult males with a diagnosis of psychopathy and adults with elevated psychopathic traits show difficulties in identifying fearful facial expressions of emotion [9–11]. Similarly, fear recognition difficulties have also been highlighted among children with conduct problems and are particularly severe among children with elevated callous-unemotional (CU) traits [12]. Moreover, psychopathic traits in adults and children are also associated with hypoactivity of the amygdala, the critical structure for the processing of threat related stimuli [13–16].

As well as fear related deficits, early descriptions of psychopathy also emphasise a pronounced lack of anxiety or nervousness [1]. Although the PCL-R bears no direct reference to low anxious or fearless–an observation that has prompted some to question the extent to which these features represent defining aspects of the disorder [17, 18]–Neumann and colleagues provide evidence that lack of fear and anxiety are comprehensively accounted for by existing PCL-R items [19]. However, psychopathic variants that are characterized by heightened levels of trait anxiety have nonetheless been identified [18, 20, 21].

The presence of such variants is highlighted in a distinction between low anxious primary psychopaths, and high anxious secondary psychopaths, in samples of adult offenders [20], as well as in juvenile developmental samples [22–24]. Results from adult samples have shown that primary psychopaths are characterized by interpersonal and affective features, while secondary psychopaths show higher trait anxiety, more borderline personality features, and more symptoms of major mental disorder [20, 22]. Furthermore, secondary variants also show greater risk scores on actuarial measures, have more criminogenic needs, and are more likely to show change through the course of treatment despite relatively similar rates of recidivism to primary psychopaths [22]. In developmental samples, it has been found that high anxious secondary CU variants show greater attention to emotionally distressing pictures [23] and higher levels of aggression and conduct problems [24]. Thus, although the high anxious variants identified in these samples do not resemble prototypical description of psychopathy [1], their identification raises the seemingly paradoxical hypothesis that while some features of the psychopathy construct are related to fear related difficulties, including impaired recognition of fearful expressions, other features may be associated with high levels of trait anxiety.

Consistent with the observation of lowered levels of anxiety in relation to psychopathic traits, there is also evidence that anxiety may protect against antisocial behaviour [25]. For example, elevated levels of anxiety and depression have been observed among males who showed a less severe pattern of low-level, chronic offending relative to those with life-course persistent or adolescence-limited antisocial behaviour [26]. Furthermore, high risk boys described as nervous and obsessional offend at lower rates in adulthood relative to other high risk males [27].

However, contrasting evidence has also been presented that fails to support the notion that anxiety protects against antisocial behaviour. A greater comorbidity of conduct problems with depression and anxiety has been identified among clinical relative to community samples [28–30], and similar relationships have been observed among adult samples [31–33]. Crucially, it has also been suggested that antisocial personality disorder (ASPD) comorbid with anxiety disorder may represent a diagnostic variant to ASPD alone [34]. Thus, the relationship between antisociality and trait anxiety remains somewhat unclear.

Although psychopathy in a forensic context is most commonly assessed using the PCL-R, psychopathic personality traits may nonetheless be observed sub-clinically using self-report personality inventories. The use of such measures is consistent with the dimensional, rather than categorical nature of the psychopathy construct [35, 36], and with the finding that psychopathic traits are normally distributed in the general population [37]. One such instrument, the Levenson Self Report Psychopathy Scale [LSRP] [38], assesses psychopathy along three distinct dimensions: callous, egocentric, and antisocial [39, 40]. The three factor solution of the LSRP is supported by findings showing differing relationships of these three dimensions with a history of violence and substance abuse, and indicators of psychopathology, in male and female convicted and college samples [39, 40].

The importance of considering the separable dimensions of the psychopathy construct have been highlighted in the finding of suppressor effects in the ways in which PCL-R Factors 1 and 2 relate to negative emotionality in offending samples [41]. Suppressor effects occur where two correlated predictors show opposing relations with a criterion variable [41]. When accounting for potential suppressor effects, convincing evidence has been found for a negative association of Factor 1 with negative emotionality, including distress, fear, and anger, while positive associations were found in relation to Factor 2 [41]. Other examples of suppressor effects in relation to discrete psychopathic traits have also been highlighted [42–44]. Thus, differing psychopathic traits may exert differential relationships with trait anxiety on the one hand, and the processing of fear related stimuli on the other.

In support of differential relationships of psychopathic traits with anxiety and fear, it has been suggested that trait anxiety and fear may be distinguishable at a conceptual level [45]. Thus, while anxiety may be defined as prolonged hypervigillance in anticipation of a non-specific threat where there is no clear danger [45], fear represents an aversive reaction to a specific threatening stimulus [46] and is associated with one of three behavioural responses in mammals: fight, flight, and freezing [47]. Furthermore, although trait anxiety is associated with a bias in selective attention toward threat related stimuli [48, 49], and an increased acquisition of fear learning [49, 50], fear and anxiety may be distinguishable at a neurobiological level in animal models of trait anxiety and fear [51–54]. However, it should be noted that this distinction remains a topic of much debate, particularly in terms of the role of sub-cortical structures including the amygdala [55]. The extent to which these constructs may share opposing relationships with discrete psychopathic traits remains relatively poorly understood.

The aim of the present studies was to assess the relationships of callous, egocentric, and antisocial psychopathic traits with trait anxiety and fear in samples of offenders and non-offenders. In Study 1 we examined the relations of distinct psychopathic traits with self-report levels of state and trait anxiety, and social anxiety and avoidance, in a sample of non-offenders. Although there is limited evidence for a gender specific test bias associated with a two-factor model of the LSRP in an undergraduate sample [56], research shows that there may be differences in the distribution of psychopathic traits between males and females [57], and that such traits are either more difficult to measure [58] or manifest differently in female samples [3]. As such, we included gender in the analysis, and tested the interactions of gender with distinct psychopathic traits for each outcome variable. In Study 2 we aimed to extend these findings using a sample of violent offenders. Furthermore, we also used a computerized test of emotional expression recognition to examine the relations of callous, egocentric, and antisocial psychopathic traits with fear recognition abilities. Across both samples it was hypothesised that state-trait anxiety and social anxiety would be positively related to antisocial traits, but negatively related to the callous and egocentric traits pertaining to the affective/interpersonal features of the psychopathy construct. With respect to emotional expression recognition in Study 2, we hypothesised that consistent with amygdala based accounts of psychopathy [7, 8, 59], offenders would show an inverse relationship of fear accuracy with callous psychopathic traits.

## Study 1

### Method

#### Participants

A total of 285 participants (87% female) were recruited to take part in an on-line questionnaire. Participants were recruited through the University’s on-line research participation scheme and were awarded course credits for taking part. Participants had a mean age of 19.1 (*SD* = 1.7) with a range of 18–35. All participants provided their written informed consent. The study was approved by the University of Birmingham, UK, Science, Technology, Engineering and Mathematics (STEM) Ethical Review Committee.

### Materials

#### Levenson Self-Report Psychopathy Scale [LSRP]

The LSRP was originally designed to parallel the two-factor solution of the PCL-R, with a primary subscale assessing a callous or manipulative interpersonal style, and a secondary subscale which tapped poor behavioural control [38]. However, the results of factor analyses of the LSRP in offending and non-offending samples revealed a best fitting three factor model, with subscales that tap the callous, egocentric, and antisocial traits of psychopathic personality [39, 40]. Although we used the full scale, 26-item version of the LSRP, the three factor solution [39, 40] utilises only 19 of the original 26 items, with the callous, egocentric, and antisocial subscales consisting of four, ten and five items respectively. Participants respond to each item on a four-point Likert scale, with responses ranging from *‘Disagree strongly’* to *‘Agree strongly’*. Adequate internal consistency and reliability for the three factor model has be demonstrated, with Cronbach’s alpha estimates of .61, .83 and .62 for the callous, egocentric and antisocial subscales [40]. Although these estimates appear low, it has been noted that the Cronbach’s alpha estimate penalises shorter scales and that inter-item correlations were found to be well with-in the recommended range [40].

#### State-Trait Anxiety Inventory [STAI]

The STAI [60] was used to measure both short-term, state feelings of anxiety [STAI-S] and also more long term anxiety as a personality trait [STAI-T]. Each subscale contains 20 items which are scored on a four-point Likert scale. State items ask a person to rate how they feel at a particular moment in time (e.g., *‘I feel tense’*) with responses ranging from *‘Not at all’* to *‘Very much so’*. Trait items enquire about a person’s feelings of anxiety in general (e.g., *‘I feel nervous and restless’*) with responses varying form *‘Almost never’* to *‘Almost always*.*’* Median alpha coefficient internal reliability estimates for the state and trait scales are reported as .93 and .90, respectively, for large independent samples of high school and college students, working adults and military personnel [60].

#### Liebowitz Social Anxiety Scale [LSAS]

Social phobia was assessed using LSAS [61]. The LSAS consists of 24 items rated on a 4-point Likert scale designed to measure fear (0 = *No fear*; 3 = *Severe fear*) and avoidance (0 = *Never avoid*; 3 = *Usually avoid*) across a variety of social interactions and performance situations. The LSAS has demonstrated acceptable internal validity, with a Cronbach’s alpha estimate of .95 for the whole scale, while the fear and avoidance subscales also demonstrate acceptable internal validity, with Cronbach’s alphas of .91 and .92 respectively [62].

#### Procedure

All measures were uploaded to an on-line survey which participants were able to access through the University on-line research participation scheme. Responses were downloaded from the survey and exported to IBM SPPS Statistics Version 20 for statistical analysis.

#### Method for analysis

We predicted that while egocentric and callous psychopathic traits would negatively predict the criterion variables, antisocial psychopathic traits would be predictive in a positive direction. To test these predictions, we used backward multiple linear regression models to investigate the association of egocentric, callous, and antisocial psychopathic traits with criterion anxiety variables. Analyses included gender (male = 0, female = 1), the callous, egocentric, and antisocial dimensions of the LSRP, and the two-way interactions of gender with each dimension (egocentric*gender, callous*gender, antisocial*gender).

### Results

Table 1 shows descriptive statistics for all participants for the LSRP callous, egocentric, and antisocial subscales, the STAI-S and STAI-T, and the SAS fear/anxiety and avoidance subscales. Scores on the LSRP callous, egocentric, and antisocial subscales were in the range of those reported in a sample of 539 undergraduate students [63]. Pearson’s correlation coefficient showed that the three subscales of the LSRP were all significantly positively correlated (all *r* > .16, *p* < .01). Furthermore, state and trait anxiety, social fear/anxiety, and social avoidance were all positively correlated (all *r* > .41, *p* < .001). To test the hypothesis that psychopathic traits differentially predict criterion anxiety variables, backward regression was performed using standardized psychopathic trait scores as predictors. Table 2 shows significant predictors from backward regression models for state and trait anxiety, social fear/anxiety, and social avoidance.

**Table 1:** Mean, standard deviation, and minimum and maximum scores for the callous, egocentric, and antisocial subscales of the LSRP, state anxiety, trait anxiety, and social fear/anxiety and avoidance scales.

	Mean (*SD*)		
	Full sample	Males	Females
LSRP Callous	4.9 (1.4)	5.7 (1.8)	4.8 (1.3)
LSRP Egocentric	19.9 (5.3)	20.7 (5.7)	19.8 (5.2)
LSRP Antisocial	10.4 (2.7)	10.3 (2.6)	10.4 (2.7)
STAI-State	37.7 (11.1)	34.8 (9.9)	38.1 (11.2)
STAI-Trait	46.0 (10.3)	44.3 (11.0)	46.2 (10.2)
SAS fear/anxiety	26.6 (11.3)	23.5 (11.3)	27.1 (11.3)
SAS avoidance	22.3 (11.6)	18.3 (8.7)	23.0 (11.9)

Note: LSRP = Levenson Self Report Psychopathy scale; STAI = State-Trait Anxiety Inventory; SAS = Social Anxiety Scale.

**Table 2:** Predictors of state anxiety, trait anxiety, social fear/anxiety, and social avoidance in an undergraduate sample.

	*β*	*SE*	*t*	*p* value
			STAI-State	
LSRP Egocentric	-1.14	.63	-1.80	.07
LSRP Antisocial	4.61	.63	7.27	< .001
		STAI-Trait	
LSRP Egocentric	-1.96	0.59	-3.35	.001
LSRP Antisocial	4.42	0.59	7.55	< .001
		SAS fear/anxiety	
LSRP Callous	-2.78	1.35	-2.06	.04
LSRP Antisocial	2.01	.67	3.00	.003
LSRP Callous x Gender	2.69	1.55	1.73	.09
		SAS avoidance	
Gender	4.60	2.01	-2.29	.02
LSRP Antisocial	1.66	.68	2.46	.02

Note: LSRP = Levenson Self Report Psychopathy scale; STAI = State-Trait Anxiety Inventory; SAS = Social Anxiety Scale.

The analysis predicting state anxiety revealed a best-fitting model with egocentric and antisocial psychopathic traits [*ΔR* ^2^ = .15, *F*(2, 284) = 26.51, *p <* .001]. We showed that egocentric and antisocial psychopathic traits predicted state anxiety in opposite directions. While antisocial psychopathic traits were a significant positive predictor of state anxiety (*β* = 4.61, *p* < .001), egocentric emerged as a negative non-significant predictor (*β* = -1.14, *p* = .07)

**Fig 1:**
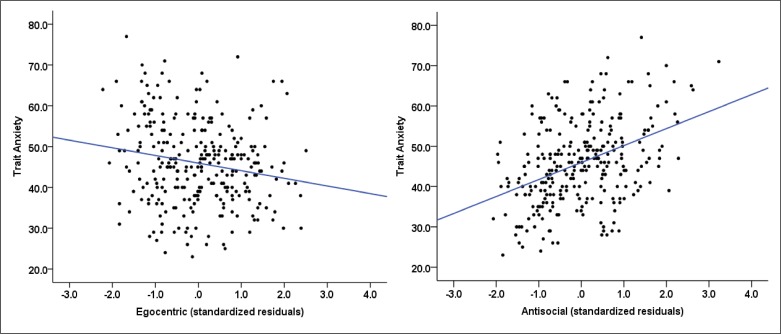
Scatter plots showing the relationship of (left) egocentric, and (right) antisocial psychopathic traits with trait anxiety in the full sample.

Consistent with our findings for state anxiety, we found a best fitting model for predicting trait anxiety that included egocentric and antisocial psychopathic traits [*ΔR* ^2^ = .17, *F*(2, 284) = 29.11, *p <* .001]. This model suggested that both egocentric (*β* = -1.96, *p* = .001) and antisocial psychopathic traits (*β* = 4.42, *p* < .001) predicted trait anxiety in opposite directions (Fig 1). Thus, while egocentric traits were a significant negative predictor of trait anxiety, we found that antisocial psychopathic traits predicted trait anxiety in a positive direction.

A best fitting model for social fear/anxiety was revealed that included callous and antisocial psychopathic traits, as well as the interaction of callousness with gender [*ΔR* ^2^ = .03, *F*(2, 284) = 3.93, *p* = .009]. We showed that callous (*β* = -2.78, *p* = .04) and antisocial (*β* = 2.01, *p* = .003) psychopathic traits were differentially predictive of social fear/anxiety, with callousness predicting lower levels of social fear/anxiety, and antisocial traits predicting social fear/anxiety in a positive direction. The final model also included the interaction of callous psychopathic traits with gender (*β* = 2.69, *p* = .09). Although the effect failed to reach significance, this suggested a less negative relationship between callous psychopathic traits and social fear/anxiety among female compared with male participants.

Finally, we found a best fitting model for the prediction of social avoidance with gender and antisocial psychopathic traits [*ΔR* ^2^ = .03, *F*(2, 284) = 5.75, *p* = .004]. This model showed that relative to males, female participants showed higher social avoidance (*β* = 4.60, *p* = .02), while antisocial psychopathic traits were found to be a significant positive predictor of social avoidance (*β* = 1.66, *p* = .02).

### Discussion

Study 1 aimed to investigate the association of callous, egocentric, and antisocial psychopathic traits with self-reported levels of state and trait anxiety, and social fear/anxiety and avoidance in a sample of non-offenders. We showed that while increasing antisocial personality traits predicted higher scores on all four anxiety measures, egocentric psychopathic traits negatively predicted both state and trait anxiety. Furthermore, we showed that although there was a significant effect of gender on social avoidance, with female participants showing higher social avoidance relative to males, there were no significant interactions of gender with psychopathic traits for any of the criterion variables.

The findings reported here support earlier findings of a positive association of antisocial behaviour with anxiety in children [25] and in adults [31, 32]. Furthermore, the finding that higher egocentric traits were associated with lower levels of state and trait anxiety is consistent with descriptions of prototypical psychopaths as lacking in nervousness or anxiety [1]. Taken together, these findings are similar to other results showing that anxiety in an incarcerated sample was negatively related to PCL-R Factor 1, but positively related to Factor 2 [21]. However, the findings that we report here suggest that while anxiety is negatively related to the egocentric features of psychopathy in a non-offending sample, there is no relationship of trait anxiety with the callous and affective features of the disorder.

Previous findings have also reported a negative association of self-reported psychopathic traits with social anxiety in a student sample [64] whereas we showed that social fear/anxiety and avoidance are positively related to antisocial traits. However, we also found a negative relationship of social fear/anxiety with callous traits. This finding suggests that the relationship of social anxiety with psychopathic traits is complex. In support of a negative relationship, others have found contrasting patterns of neural activity in the amygdala of psychopathic offenders compared with patients with social phobia during an aversive conditioning task [65]. These authors showed that while social phobics displayed hyperactivity of the amygdala, hypoactivity was observed among psychopathic offenders [65]. These results suggest that psychopathy and social phobia may represent diametric opposites in terms of amygdala function.

Although non-significant, the relationship of callous psychopathic traits with social fear/anxiety also appeared to differ between male and female participants, with a less negative relationship observed in female compared with male participants. The inclusion of a gender interaction is consistent with the suggestion that psychopathic traits may be differentially distributed, or manifest differently, in male and female samples [3, 57, 58].

Although the findings of Study 1 provide further support for differential associations of psychopathic traits with self-report levels of anxiety and social anxiety, it remains to be seen whether similar relationships can be identified in an offending sample. Furthermore, these results fail to shed new light on the possibility that anxiety and fear may be differentially related to distinct psychopathic traits in offending samples. In Study 2 we examined the relationships between psychopathic traits and anxiety constructs in a sample of incarcerated violent offenders. We also used a facial affect recognition task as an indicator of the capacity to recognize fear face affect.

## Study 2

### Method

#### Participants

We recruited an offending sample of 29 violent offenders ranging in age from 24 to 62 years, (*M* = 44.15, *SD* = 8.2). Violent offenders were recruited from and tested at the Therapeutic Community for adult male prisoners at HM Prison Grendon, UK, where they were incarcerated for violent offences, as defined to include: murder, wounding with the intent to do grievous bodily harm, and the rape or attempted rape of an adult or child victim [66]. While 16 participants had committed an offense including murder or wounding with intent, 13 participants had committed a violent offense with a sexual element, including the rape or attempted rape of an adult or child. A high proportion of participants had participated in treatment programs including Enhanced Thinking Skills (*n* = 19), aimed at developing new ways of thinking about people and problems. The majority of sexual offenders had completed the Sex Offender Treatment Program (*n* = 11). Roughly half of the sample reported a history of early physical and/or emotional abuse (*n* = 16). All participants provided their written informed consent. The study received ethical approval from the University of Birmingham, UK, Science, Technology, Engineering and Mathematics (STEM) Ethical Review Committee, and was approved by the UK National Offender Management Service (NOMS).

#### Materials

We used the LSRP, STAI, and SAS for the assessment of psychopathic traits, state-trait anxiety, and social anxiety, respectively (see Study 1). All participants completed the LSRP and the SAS. Three participants failed to complete the STAI-S and the STAI-T. For the emotion recognition task ten different White models (five females) were selected from the NimStim Face Stimulus Set (http://www.macbrain.org/resources.htm) [67]. Stimuli included each model showing seven different expressions: neutral, angry, disgust, fear, happy, sad, and surprise. Models were selected based on the NimStim validity data that indicate a high mean proportion correct for each expression (mean proportion correct, standard deviations in brackets): angry = .85 (.13), disgust = .85 (.13), fear = .84 (.13), happy = .85 (.13), sad = .85 (.13), surprise = .85 (.13), neutral = .84 (.13) [67]. In order to manipulate the intensity of the emotional expressions, each expression was morphed from neutral to 100% expressive in ten successive frames using the STOIK Morph Man software (http://www.stoik.com/products/video/STOIK-MorphMan/). This resulted in ten morphed continua for each expression for the ten selected models. For task purposes, we selected three frames of varying intensity for each expression: mild intensity (10% expressive), moderate intensity (55% expressive), and normal intensity (90% expressive). Standard neutral expressions were not presented. Thus we had 18 faces across all expressions for each model, 180 faces in total.

#### Procedure

Offenders were tested individually in a quiet room on the prison wing. Facial expression stimuli for the emotion recognition task were presented using E-Prime 2.0 stimulus presentation software on a Samsung Electronics laptop computer. Faces were presented in a random order and remained on screen until the participant responded. Participants were asked to categorize faces as one of the seven core emotions: neutral, angry, disgust, fear, happy, sad, or surprise, using the numeric keys 0–6 respectively. The expression labels were listed on the left hand side of the screen alongside the relevant number key. There were 180 trails, each presenting a different face varying in sex, expression and intensity. All participants completed the emotion recognition task followed by pen-and-paper versions of the self-report inventories. In this paper we concentrate on the ability to recognize moderate and normal intensity expressions only, given the neutral appearance of the lowest intensity expressions. A comparison of accuracy between offenders and non-offenders is beyond the scope of this paper, which aims to examine distinct relations of fear recognition with psychopathic traits.

#### Method for analysis

We used backward linear regression models to examine the extent to which callous, egocentric, and antisocial psychopathic traits were predictive of state and trait anxiety, and social fear/anxiety and avoidance. For analyses of accuracy of emotional expression recognition, we concentrated on the number of correct responses for each emotion summed across moderate (55% expressive) and high (90% expressive) intensity expressions.

### Results

Mean scores (standard deviation in parentheses) for offending participants on all scales were as follows: egocentric = 16.2 (5.3); callous = 7.4 (3.1); antisocial = 11.5 (3.8); state = 31.4 (11.4); trait = 42.0 (10.1); social fear/anxiety = 28.6 (15.8); social avoidance = 22.0 (12.8). Pearson’s correlation coefficient revealed significant positive interrelationships between the three subscales of the LSRP (*r* > .46, *p* < .05). Furthermore, there were positive correlations between state and trait anxiety, social fear/anxiety, and social avoidance (*r* > .64, *p* < .001). However, we failed to observe any significant relationships of fear accuracy with state, trait anxiety, social fear/anxiety, or avoidance (*r* = -.00 - .02, *p* = *ns*). These results suggest that anxiety and fear recognition represent independent constructs that may be differentially related to distinct psychopathic traits. To test the hypothesis that psychopathic traits differentially predict criterion anxiety/fear variables, backward regression was performed using standardized psychopathic trait scores as predictors. Table 3 shows significant predictors from backward regression models for state and trait anxiety, social fear/anxiety, and social avoidance, as well as performance on the emotion recognition task.

**Table 3:** Predictors of state anxiety, trait anxiety, social fear/anxiety, and social avoidance in an offending sample.

	*β*	*SE*	*t*	*p* value
		STAI-State		
LSRP Callous	4.90	1.97	2.49	.02
		STAI-Trait		
LSRP Egocentric	-4.45	2.00	-2.23	.04
LSRP Antisocial	7.16	2.10	3.42	.002
		Fear recognition		
LSRP Callous	-2.02	0.89	-2.28	.03

Note: LSRP = Levenson Self Report Psychopathy scale; STAI = State-Trait Anxiety Inventory.

**Fig 2:**
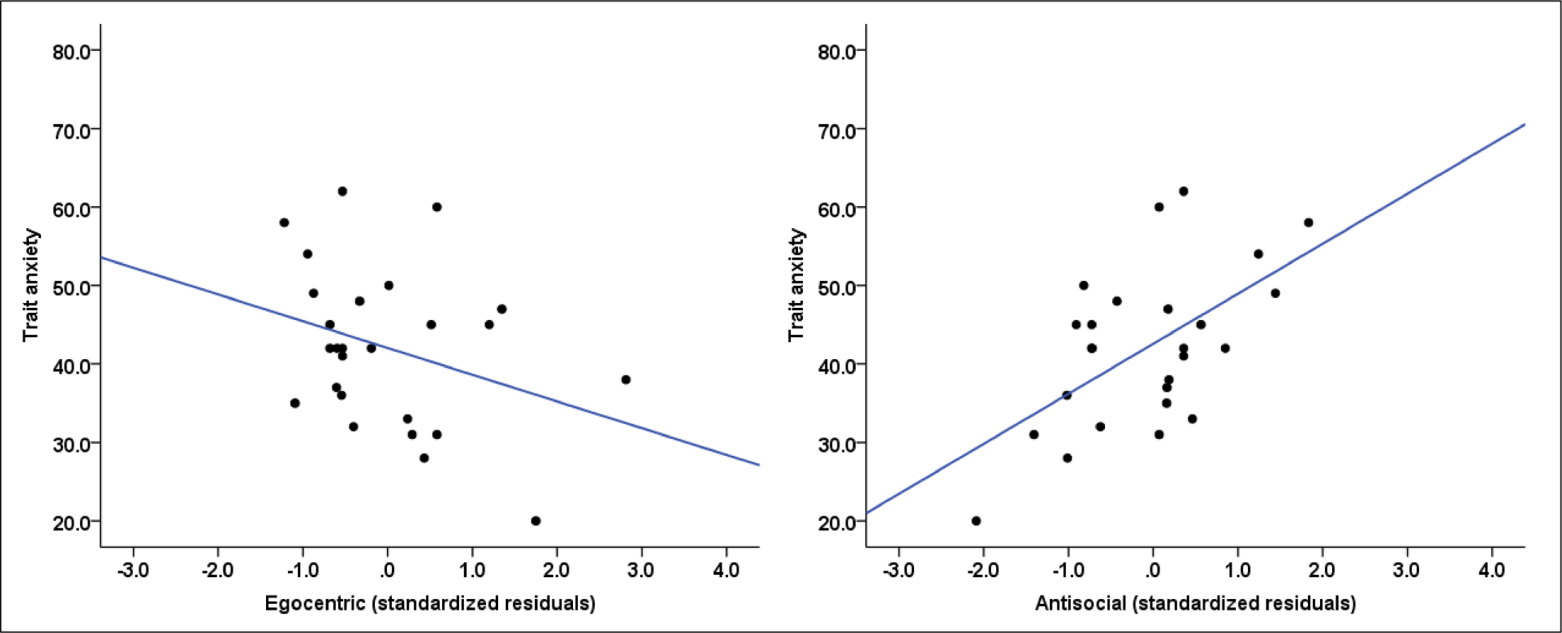
Scatter plots showing the relationship of (left) egocentric, and (right) antisocial psychopathic traits with trait anxiety in the violent offender sample.

Backward regression showed that callous psychopathic traits solely predicted state anxiety [*ΔR* ^*2*^ = .17, *F*(1, 25) = 6.19, *p <* .05], with higher levels of callous psychopathic traits predictive of elevated state anxiety scores (*β* = 4.90, *p* < .05). A best fitting model also showed that antisocial and egocentric psychopathic traits predicted trait anxiety scores in opposite directions [*ΔR* ^*2*^ = .28, *F*(2, 25) = 5.94, *p <* .01]. While antisocial traits positively predicted trait anxiety (*β* = 7.16, *p* < .01), egocentric predicted trait anxiety in a negative direction (*β* = -4.45, *p* < .05) (Fig 2). These results are consistent with the findings of Study 1 and show distinct relations of psychopathic traits with trait anxiety. The regression model for predicting social fear/anxiety was non-significant [*ΔR* ^*2*^ = .07, *F*(1, 28) = 2.96, *p =* .10], and none of the predictor variables satisfied criteria for model inclusion for social avoidance.

**Fig 3:**
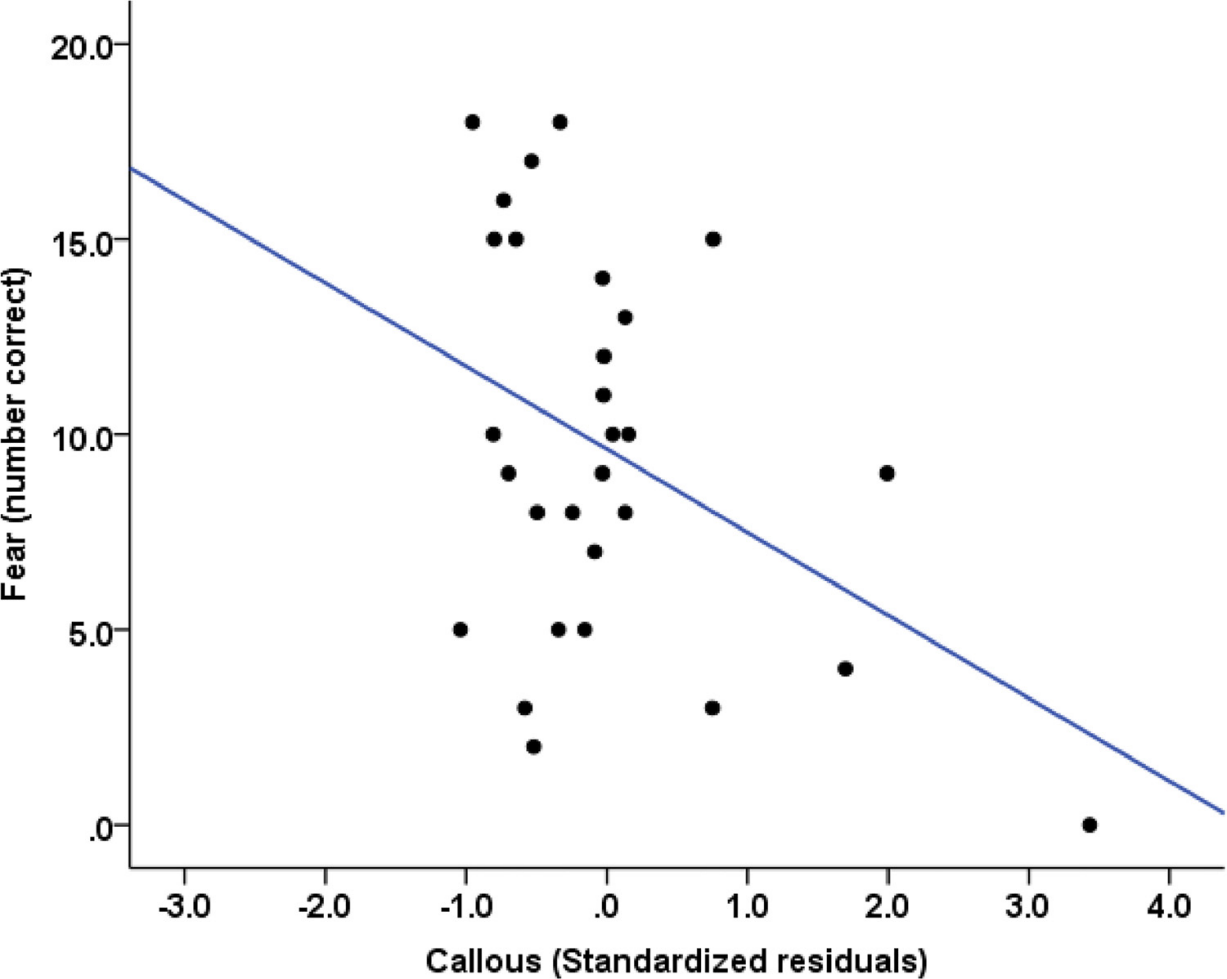
Scatter plot showing the relationship of callous psychopathic traits with accuracy of fearful face recognition.

However, consistent with our hypothesis, we showed that fear accuracy on the emotion recognition task was solely predicted by callous psychopathic traits [*ΔR* ^*2*^ = .13, *F*(1, 28) = 5.17, *p <* .05], with higher callous scores predicting more severely impaired fearful face recognition (*β* = -2.02, *p* < .05) (Fig 3). These results suggest that although antisocial psychopathic traits were positive predictors of anxiety, fearful face recognition was nonetheless negatively predicted by other aspects of the psychopathic personality. None of the predictor variables satisfied inclusion criteria for models predicting anger, disgust, happy, sad, or surprise expressions.

### Discussion

Study 2 aimed to extend upon the findings of Study 1 in an offending sample, and to test the additional hypothesis that different aspects of the psychopathy construct would show distinct relations with fearful face recognition. Specifically, we hypothesised that antisocial features would be positively related to anxiety constructs, that egocentric features would be negatively related to anxiety constructs, and that callous traits would be associated with a greater impairment in fear face affect recognition. Results were largely consistent with those from Study 1. Antisocial traits positively predicted state and trait anxiety, egocentric traits negatively predicted trait anxiety, while callous traits predicted more impaired fear recognition. This finding is consistent with results showing difficulties in recognizing fearful face affect among children with CU traits, adults with high psychopathic traits, and adult male psychopaths [9–12, 14] (also see [68, 69] for meta-analyses) and suggests that these problems may be related to the callous lack of empathy that differentiates psychopaths form more generally antisocial individuals. Crucially, we observed no relationship of psychopathic traits with recognition accuracy for other expressions, suggesting a fear specific relationship with callous traits. Furthermore, there was no significant association of fear accuracy with any of the anxiety constructs.

It is suggested that the original two-factor structure of the LSRP is not sensitive to a lack of empathy or fear [38], both of which are associated with amygdala dysfunction [59]. However, the findings of the present study show that the three-factor structure callous subscale is sensitive to impaired recognition of fear face affect in an offending sample. These findings indicate that the callous subscale may be more sensitive than the original primary psychopathy scale to psychopathy related impairments in the processing of fear related stimuli.

We also found an unexpected, positive relationship of callous traits with state anxiety, suggesting that higher callous individuals experienced some state like feelings of negative affect at the time of completing the study. While the callous subscale in this study was sensitive to problems in fearful face recognition, and this presumably reflects poor amygdala function [59], it has been highlighted that some social threats may induce anxiety independent of the amygdala [70]. Thus, individuals with fear deficits may nonetheless be sensitive to anxiety in relation to social threats. However, it is possible that the relationship of callous traits with state anxiety observed here may reflect increased levels of frustration. It is argued by Blair [71] that problems in stimulus-reinforcement learning and reversal learning in psychopathy may leave these individuals at increased risk of frustration, where expected goals are not achieved through engaging in a particular action [71]. However, there is limited evidence for elevated frustration in relation to psychopathic tendencies. Furthermore, the extent to which such frustration based increases in negative affect may be detected using the STAI state anxiety scale is unknown.

## General Discussion

The present article summarises the results from two studies that aimed to address the relationship of callous, egocentric, and antisocial psychopathic traits with state-trait anxiety, social anxiety, and fear recognition. In Study 1 we found positive relationships of antisocial psychopathic traits with state and trait anxiety, and with the fear/anxiety and avoidance dimensions of social anxiety. These findings help to clarify the relationship of antisocial traits with the propensity to experience feelings of anxiety. We also showed that egocentric psychopathic traits were negatively related to both state and trait like anxiety. Study 2 extended these findings to an offending sample. Here we showed that antisocial traits predicted heightened feelings of state and trait anxiety, while egocentric traits predicted trait anxiety in the negative direction. Furthermore, callous traits predicted reduced fear accuracy, and unexpectedly, showed a positive relationship with state anxiety. Notably, psychopathic traits were unrelated to the recognition of other emotions besides fear, and there was no relationship of fear accuracy with any of the criterion anxiety variables.

The finding of distinct relations of psychopathic traits with trait anxiety are consistent with earlier findings in relation to PCL-R Factors 1 and 2 and negative emotionality more generally [41, 72–74]. Although distinct relations of PCL-R Factors 1 and 2 with scores on the Welsh Anxiety Scale [WAS] [75] have been observed [21], it has been suggested that the WAS may measure negative affectivity more generally [76], rather than a specific propensity to feel anxiety. Similarly, distinct and opposite relations of PCL-R Factors 1 and 2 with negative affectivity, including emotional distress, fearfulness, and anger hostility, have been observed in a large sample of male prisoners [41]. The results of Studies 1 and 2 therefore contribute to a more refined understanding of the psychopathy-anxiety relationship in both samples of offenders and non-offenders, and build on previous findings relating to negative affect more generally.

These findings support the notion that low anxiety does not necessarily protect against antisocial behaviour, and may support the existence of psychopathic variants. Such variants, termed primary and secondary, are distinguishable on the basis of anxiety levels and have been differentiated in both adult [20, 77, 78] and developmental [23, 24] samples. For example, Kimonis and colleagues report that high anxiety variants of children with elevated CU traits show greater attention to negative emotional stimuli compared with their low anxious counterparts [23]. These results indicate that high and low anxiety variants of CU traits may be distinguishable on the basis of emotion processing differences and suggest different etiological pathways to elevated psychopathic traits.

More recent evidence for psychopathic subtypes in adult male offenders suggests the presence of three distinct variants of extreme scorers on the PCL-R: two of these variants presented as ‘true’ psychopaths, and were termed manipulative and aggressive types, while a third variant, termed sociopathic, was found to be conceptually more similar to ASPD [79]. The presence of high anxiety antisocial offenders may have implications for clinical practice in a forensic setting. For example, it has been suggested that meditative techniques that affect the functioning of neural regions involved in the cortical control of emotion may be of use with offenders who show emotion regulation difficulties [80]. The use of such techniques in a forensic context with more generally antisocial offenders may help to relieve the anxiety and negative affective states that characterize these individuals.

The finding of a negative association between callous psychopathic traits and fear recognition adds to a considerable body of evidence highlighting impaired processing of fearful face affect in psychopathic personality [68, 69]. In support of a negative relationship of callous traits with fear processing, CU traits are inversely associated with amygdala responsivity to fearful expressions in adolescent samples [14, 15, 16], and amygdala responsivity to fearful faces mediates a link between CU traits and proactive aggression [81].The finding reported here of a negative relationship of callous traits with fear recognition did not extend to other emotions, and suggests a fear specific relationship of callous traits with emotion processing difficulties. These findings are also consistent with results showing that the affective facet of psychopathy predicts lower skin conductance responses among psychopathic offenders during the acquisition of aversive stimulus-reinforcement associations [82]. However, it is important to note that although impaired fearful face recognition may be indicative of dysfunction in the neural architecture for fear, it may not represent a reliable indicator of the ability to experience feelings of fear *per se* [83].

Our results highlight a seemingly paradoxical relationship of psychopathic traits with anxiety and fear: while antisocial traits were related to elevated anxiety on the one hand, callous traits were related to impairments in recognizing emotional expressions of fear on the other. This paradox may, however, be resolved when considering conceptual differences between anxiety and fear, as well as differences in the neurobiological underpinnings of these two constructs [45]. In rodent models of anxiety and fear, ablation of specific neural projections selectively impairs anxiety and fear responses, and supports the idea that these responses are distinct [84]. Furthermore, in Study 2 we found no evidence for a positive association of state or trait anxiety with accuracy of fear face recognition. However, the distinction between anxiety and fear remains a topic of much debate (see [55]), and the precise relationship of these responses with distinct psychopathic traits remains an important question.

In interpreting the findings of Studies 1 and 2 it should be noted that a deceitful and manipulative interpersonal style represents a hallmark feature of psychopathic personality and that these traits may be linked with dishonest responding on self-report measures of psychopathy [85]. Furthermore, the limited number of items that make up the callous and egocentric subscales of the LSRP may limit the extent to which these subscales represent reliable measures of these constructs. Despite these difficulties, findings from a meta-analysis suggest that self-report psychopathy scales are valid indicators of psychopathic traits [86]. In future research with clinical and offending populations, the PCL-R assessment of psychopathy, which includes file review of collateral information, may help to navigate the potential pit-falls associated with the self-report measurement of psychopathy.

Also, findings from Study 1 may have been influenced by low levels of psychopathic traits in the general population. For example, a weighted prevalence for ‘possible’ psychopathy of 2.3% has been noted in a representative sample of the general household population of Great Britain [57]. Thus, although similar relationships between psychopathic traits and anxiety were also observed in Study 2 in an offending sample, generalizations to clinical forms of psychopathy should be made with considerable caution. Also, while the findings form Study 1 controlled for the influence of gender, the extent to which the findings from Study 2 with adult male offenders generalize to female offending samples remains unknown.

In conclusion, callous, egocentric, and antisocial psychopathic traits show distinct relations with measures of anxiety and fear. The finding of distinct and opposite relations of egocentric and antisocial traits with trait anxiety underlines the importance of considering the multifaceted nature of the psychopathy construct, and may support the existence of high anxiety extreme psychopathy scorers. Such individuals who lack the core affective features of psychopathy may also be labelled ‘sociopathic’ [79]. The finding that fearful face recognition was impaired among high callous traits offenders suggests that although some antisocial offenders may show high anxiety, those with elevated callous traits may nonetheless show impaired functioning of the circuitry for fear. Such fear related deficits have been consistently indexed by CU traits in both developmental and adult samples. Differential relationships of distinct psychopathic traits with anxiety and fear also lends support to the argument that anxiety and fear represent separable constructs that are distinguishable at the conceptual and the neurobiological level. Furthermore, our results show support for models of psychopathy that emphasise the importance of distinct traits.

## Supporting Information

S1 File Study 1 minimal dataset. (XLSX)

S2 File Study 2 minimal dataset. (XLSX)
